# An efficient method for visualization and growth of fluorescent *Xanthomonas oryzae *pv. *oryzae in planta*

**DOI:** 10.1186/1471-2180-8-164

**Published:** 2008-09-30

**Authors:** Sang-Wook Han, Chang-Jin Park, Sang-Won Lee, Pamela C Ronald

**Affiliations:** 1 Department of Plant Pathology, University of California, One Shields Avenue, Davis, CA., 95616, USA

## Abstract

**Background:**

*Xanthomonas oryzae *pv. *oryzae*, the causal agent of bacterial blight disease, is a serious pathogen of rice. Here we describe a fluorescent marker system to study virulence and pathogenicity of *X. oryzae *pv. *oryzae*.

**Results:**

A fluorescent *X. oryzae *pv. *oryzae *Philippine race 6 strain expressing green fluorescent protein (GFP) (PXO99_GFP_) was generated using the *gfp *gene under the control of the neomycin promoter in the vector, pP*neo*-*gfp*. The PXO99_GFP_strain displayed identical virulence and avirulence properties as the wild type control strain, PXO99. Using fluorescent microscopy, bacterial multiplication and colonization were directly observed in rice xylem vessels. Accurate and rapid determination of bacterial growth was assessed using fluoremetry and an Enzyme-Linked ImmunoSorbant Assay (ELISA).

**Conclusion:**

Our results indicate that the fluorescent marker system is useful for assessing bacterial infection and monitoring bacterial multiplication *in planta*.

## Background

*Xanthomonas oryzae *pv. *oryzae*, a yellow-pigmented Gram-negative bacterium, is the causal agent of bacterial blight disease of rice (*Oryzae sativa *L.) plants. *X. oryzae *pv. *oryzae *infection can cause yield loss of up to 50% in tropical Asia [1]. *X*. *oryzae *pv. *oryzae *infects rice leaves through natural openings such as hydathodes and/or wounded sites and then primarily colonizes the vascular tissues by propagating in the xylem. Increased extracellular polysaccharide secretion accompanies bacteria growth, eventually causing a block in the vascular system [2]. The early symptoms therefore start with wilting in the infected leaves and enlargement in length and width of the legions of leaf blight [3]. As the symptom progress, severe necrosis occurs along the interveinal regions. Eventually, the whole leaf becomes whitish and greyish, and then dies [2,4]. Over the last few decades, the challenge in elucidating biological phenomena has been met by advances in techniques, which have accelerated our understanding of biological events. In particular, useful tools have been developed to evaluate cellular dynamics *in vivo*. One of the best examples of a technique that has facilitated cell-based studies is marker systems that use fluorescent proteins (FPs). Since the green fluorescent protein (GFP) was first discovered from the jellyfish *Aequorea victoria *in 1962 [5], various types of FPs, including red, yellow, and cyanine fluorescent proteins, have been developed and used in fields such as biophysics, biochemistry, and plant pathology [6-8]. The proteins are stable [9], non-species specific [10,11], and have no requirement of specific substrate [11]. Therefore, labelling a specific target protein with a FP is a powerful molecular tool for a cell biology study since it provides the ability to visualize, track, and quantify targets in living cells with high spatial and temporal resolution essential features for understanding biology systems [8]. In addition, bimolecular fluorescence complementation (BiFC) analysis using split-FP systems has been successfully applied to determine protein-protein interactions *in planta *as well as in animals [12,13]. Recently, FPs have been used for monitoring living organisms such as *Lactobacillus sakie*, *Pseudomonas syringae*, *X. axonopodis *pv. *dieffenbachiae*, and *Xylella fastidiosa *in their hosts [14-17].

To determine virulence and pathogenicity of bacterial pathogens in plant-microbe interactions, researchers typically quantify bacterial multiplication *in planta *by counting the number of bacterial colonies in plate assays of leaf extracts [18,19]. This method consists of leaf harvesting, tissue maceration, colony plating, incubating, and counting of bacterial cells. These methods are time-consuming and labour intensive, especially for slow-growing bacteria like *X. oryzae *pv. *oryzae*. In addition, the results frequently show large amounts of variation depending on experimental conditions.

Here, we provide an improved method for the study of *X. oryzae *pv. *oryzae *using a fluorescent marker system. It not only eliminates many of the difficulties of conventional methods but also allows for reliable and rapid monitoring of bacteria *in planta *prior to the formation of symptoms.

## Results and discussion

### Generation of *X. oryzae *pv. *oryzae *expressing GFP

**Figure 1:**
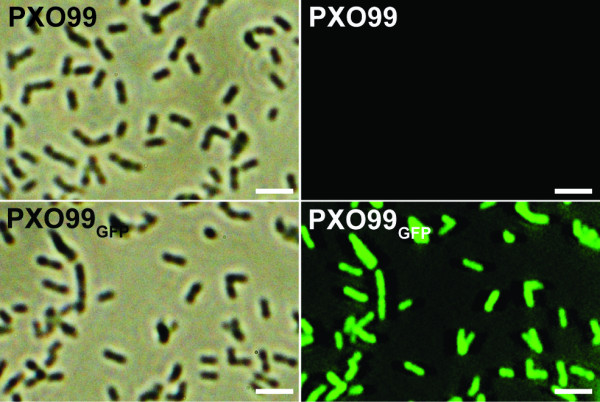
**Observation of *Xanthomonas oryzae *pv. *oryzae *carrying green fluorescence protein**. PXO99_GFP _selected on PSA plates containing kanamycin (50 μg/ml) were cultured with 5 ml PS broth medium, harvested with centrifugation, washed with distilled water three times, re-suspended with 10 μl of autoclaved water, and then observed with microscope equipped with a fluorescein isothiocyanate filter (excitation filter, 450 to 490 nm; emission filter, 520 nm; dichroic mirror, 510 nm). Lefts are images with PXO99 wild type (up) and PXO99_GFP _(down) strains under bright light. Rights are the images of the bacteria with illumination of UV. The bars in the left bottom of each image indicate 5 μm.

*X. oryzae *pv. *oryzae *Philippine race 6 (PXO99) carrying the *gfp *gene was generated using pP*neo*-*gfp*, which is a new construct based on the broad-host-range probe vector, pPROBE-*gfp *[20] and the pML122 vector [21]. The pPROBE-*gfp *plasmid was previously used as a marker to assess expression of genes of interest in *Xyllela festidosa *and *X. campestris *pv. *campestris *[22]. The neomycin promoter in pML122 was successfully used for protein expression in *X. oryzae *pv. *oryzae *[23]. To generate a plasmid, pP*neo*-*gfp*, which is approximately 400 nucleotides long and contains the neomycin promoter, was taken from the pML122 plasmid and ligated into a pPROBE-*gfp *vector. The modified construct was introduced into PXO99 wild type strain and transformants were selected on peptone sucrose agar (PSA) plate containing kanamycin (50 μg/ml) for selection of pP*neo*-*gfp*. Expression of GFP in the transformants was tested by Western-blot analysis using an anti-GFP antibody (data not shown). *X. oryzae *pv. *oryzae *carrying pP*neo*-*gfp *(PXO99_GFP_) showed strong fluorescence (Fig. 1). Our results demonstrate that the PXO99_GFP _strain carrying the pP*neo*-*gfp *vector constitutively and strongly expresses GFP in *X. oryzae *pv. *oryzae*, suggesting that the marker system might be applied to study plant-microbe interactions *in planta*.

### The PXO99_GFP _strain is useful for studies of *X. oryzae *pv. *oryzae *pathogenesis

**Figure 2:**
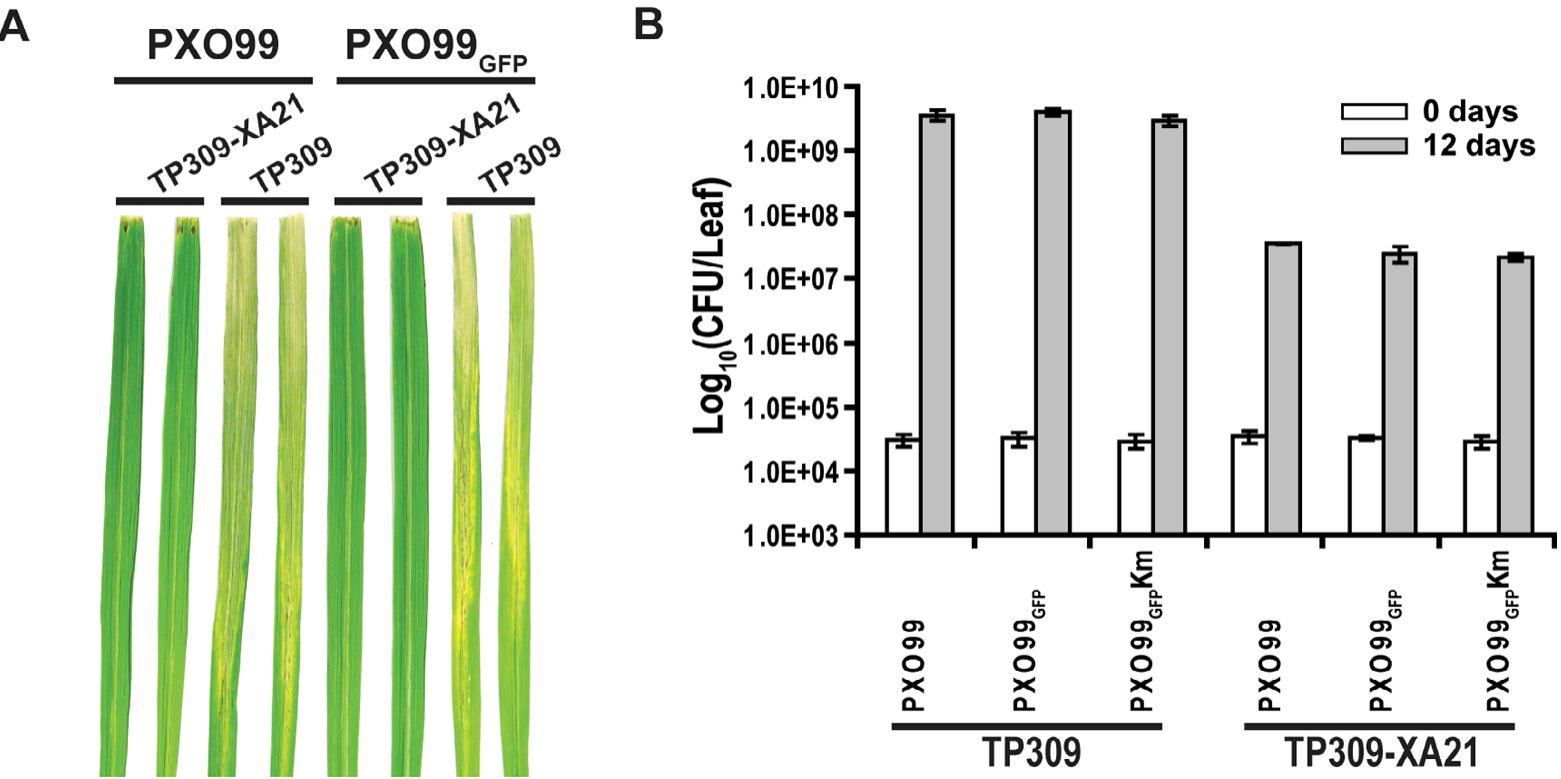
**Pathogenicity and virulence test for PXO99_GFP _*in planta***. A. Water-soaked disease lesions on two leaves from TP309 and TP309-XA21 at 12 days after PXO99 or PXO99_GFP _strains inoculation. B. After culture of PXO99 and PXO99_GFP _strains on PSA plates containing cephalexin, cells were diluted to 1.0 × 10^8 ^CFU/ml, and then inoculated onto rice leaves of TP309 (susceptible) and TP309-XA21 (resistant) lines using the scissor clipping method. Bacteria were recovered from the leaves at 0 (white bar) and 12 (grey bar) DAI and serially diluted, and then spread on PSA plates containing either cephalexin or cephalexin/kanamycin for colony counting. Each bar represents averages ± standard deviation of three sampled leaves per treatment. The experiments were repeated three times with more than ten rice leaves from three individuals each time. Km, kanamycin.

First, we examined whether the pP*neo*-*gfp *vector/plasmid had an affect on the pathogenicity and virulence of *X. oryzae *pv. *oryzae *in rice. The leaves from *japonica *rice varieties Taipei 309 (TP309, susceptible to PXO99) and a transgenic line of TP309 expressing the XA21 resistance protein (TP309-XA21, resistant to PXO99) were inoculated with the PXO99 or PXO99_GFP _strains using the scissor clipping method [24]. The typical inoculation symptoms were consistent between the PXO99 and PXO99_GFP _strains. In the susceptible line (TP309), lesions that developed were approximately 17 to 23 cm in length at 12 DPI, while lesions were less than 1 cm in the resistant line (TP309-XA21). (Fig 2A) These results suggest that pP*neo*-*gfp *introduction into the PXO99 strain does not affect pathogenicity.

These results were also confirmed with bacterial growth *in planta*. To determine the multiplication of the PXO99 and PXO99_GFP _strains *in planta*, the bacteria were recovered from infected rice leaves at 12 DAI, and dotted on PSA plates. Colonies were counted on PSA plates containing cephalexin or kanamycin, an antibiotic for selection of *Xanthomonas *spp. (Fig. 2B). Cephalexin is appreciated as an antibiotic for selection of *Xanthomonas *[25]. Populations of the susceptible lines of both PXO99 and PXO99_GFP _strains increased by 3.7 × 10^9 ^and 4.1 × 10^9^, respectively, and both strains of the resistant lines increased by 3.2 × 10^7 ^and 2.7 × 10^7^, respectively. These results indicate that expression of GFP in the bacterium, *X. oryzae *pv. *oryzae*, has no effect not only in the phenotypic symptom but also on bacterial growth *in planta*.

Bacteria have a tendency to lose exogenous plasmids in the absence of antibiotics [26]. We observed that pP*neo*-*gfp *in the PXO99 strain was not stable under non-selective conditions on PS medium without kanamycin (data not shown). Population increase was therefore measured on PSA plates containing cephalexin/kanamycin (PXO99_GFP_Km in Fig. 2B) to examine the stability of pP*neo*-*gfp *under host conditions (Fig. 2B). Surprisingly, the populations of the PXO99_GFP _strain at 12 days did not vary with or without the kanamycin antibiotics, indicating that the pP*neo*-*gfp *plasmid is stable during propagation under host conditions. Recently, several reports showed similar phenomena that bacterial pathogens carry plasmid-borne fluorescent proteins in hosts. *L. delbrueckii *ssp. *lactis *and *L. fructosus *expressing GFP on plasmid in chickens allowed to trace it in the gastro-intestinal tract using fluorescence microscopy [15]. Another notable examples is *X. axonopodis *pv. *dieffenbachiae *strain containing p519ngfp plasmid [14]. This strain still showed and the fluorescence in the host, anthurium, even at 5 weeks after inoculation [14]. Taken together, although it is poorly understood why bacterial pathogens carry exogenous plasmids under host conditions, plasmid-borne GFP should be useful tool for tracing pathogen in the host.

In order to investigate if there is a difference in the disease progress between the PXO99 and PXO99_GFP _strains, bacterial growth curves and lesion length development were established at 0, 4, 8, and 12 DAI [see Additional file 1]. Lesion lengths and bacterial populations at 4 and 8 DAI were nearly identical between the strains, indicating that the continuously expressed GFP did not affect disease progress. Tsuge, et al., reported that bioluminescent transconjugants of *X. oryzae *pv. *oryzae *were indistinguishable in pathogenicity with the parental strain [27]. To date, continuous fluorescence in bacteria did not alter the pathogenicity and virulence in the hosts. To apply our system with other patho-system, the stability of pP*neo*-*gfp *plasmid must be checked *in planta *growth.

We also confirmed the pathogenicity and virulence of the PXO99_GFP _strain and the stability of pP*neo*-*gfp *in the PXO99_GFP _strain using resistant and susceptible lines of Kitaake, another *japonica *cultivar, that has emerged as a model variety for rice genomics analysis [28]. The results from the Kitaake lines were consistent with the results from TP309 lines, showing that PXO99_GFP _strain had no detectable affect on the pathogenicity and virulence of the bacteria. These results suggest that the PXO99_GFP _strain can be used with various cultivars. Taken together, our results indicate that the fluorescent *X. oryzae *pv. *oryzae *PXO99_GFP _strain with the pP*neo*-*gfp *plasmid is a useful tool for studies of pathogenesis.

### Visual observation of PXO99_GFP _*in planta*

**Figure 3:**
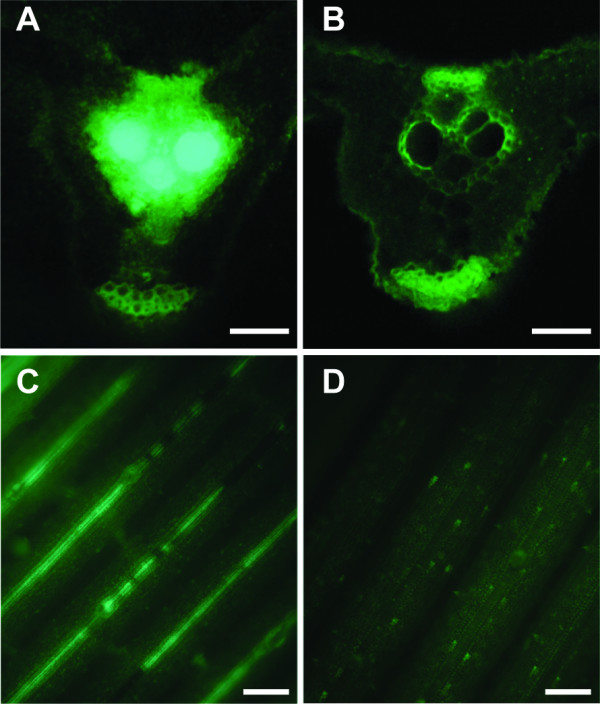
**Visualization of *X. oryzae *pv. *oryzae *expressing GFP *in planta***. TP309 (A and C) and TP309-XA21 (B and D) were inoculated with PXO99_GFP_, harvested at 12 DAI, and then visualized under Zeiss Axiophot fluorescence microscope (Jena, Germany). Transverse sections (A and B) and leaf surface (C and D) were observed with excitation from 450 to 490 nm and emitted light collected at 520 nm at × 40 (A and B) and × 10 (C and D) magnification. Bars in A and B, 50 μm. Bars in C and D, 10 μm.

We examined if the multiplication and colonization of bacteria can be visualized in rice leaf tissue. TP309 and TP309-XA21 were inoculated with the PXO99_GFP _strain, and then at 12 DAI a small segment of the leaf five centimetres down from the inoculation site was harvested. The leaf tissue was cut into segments with a razor blade, and the transverse sections of the leaf were observed under a fluorescent microscope (Fig. 3A and 3B). Fluorescence was detected at inoculation sites of both TP309 and TP309-XA21 (data not shown). As expected, no fluorescent signal was observed in the xylem of the TP309-XA21 lines at the site apart from infection sites since the PXO99_GFP _strain could not spread through vascular systems in TP309-XA21 (Fig. 3B). On the other hand, the PXO99_GFP _strain propagated along vascular systems and was clearly observed in those of TP309 lines at the same position (Fig. 3A). These results demonstrate that the PXO99_GFP _strain proliferated in the susceptible lines but not in resistant lines and that the difference can easily be detected using fluorescent microscopy. Our observation of PXO99_GFP _in the xylem is consistent with the findings of previous studies showing that *X. oryzae *pv. *oryzae *is a vascular pathogen [2,3].

To test if PXO99_GFP _xylem colonization is detectable at the leaf surface without using a thin section, we examined the surface of the leaf every five-centimetres from the inoculation site (Fig. 3C and 3D). The fluorescent PXO99_GFP _strain was detected along the entire length of the leaf of susceptible TP309 lines in both vertical and transverse vascular bundles, even in symptomless regions that are 30 cm away from the inoculation site at 12 DPI (data not shown). However, the fluorescence from the PXO99_GFP _strain in the resistant line, TP309-XA21, was not detected even in the first five-centimeter region from inoculation site (Fig. 3D). This data shows that GFP can be used for efficient and rapid detection of *X. oryzae *pv. *oryzae in planta*. Additionally, we tested bacterial migration along vascular system in TP309 and TP309-XA21 lines at earlier time (4 DAI). Although lesion lengths were almost similar in both lines at 4 DAI, bacterial migrations were clearly different. PXO99_GFP _strain migrated 8.6 cm and 2.2 cm from inoculation sites in TP309 and TP309-XA21, respectively. These observations indicate that multiplication and localization of *X. oryzae *pv. *oryzae *in the rice vascular system occurs before typical disease symptoms, such as wilting and yellowing of leaf blades, can be detected [see Additional file 2]. In other words, although disease symptoms are not clearly apparent at 4 DPI [see Additional file 1], bacterial localization can be determined [see Additional file 2]. This result demonstrates that the PXO99_GFP _strain can be used to obtain an earlier diagnosis of infection compared to conventional methods.

### Quantification of PXO99_GFP _strain using a fluoremeter or enzyme-linked immunosorbent assay

**Figure 4:**
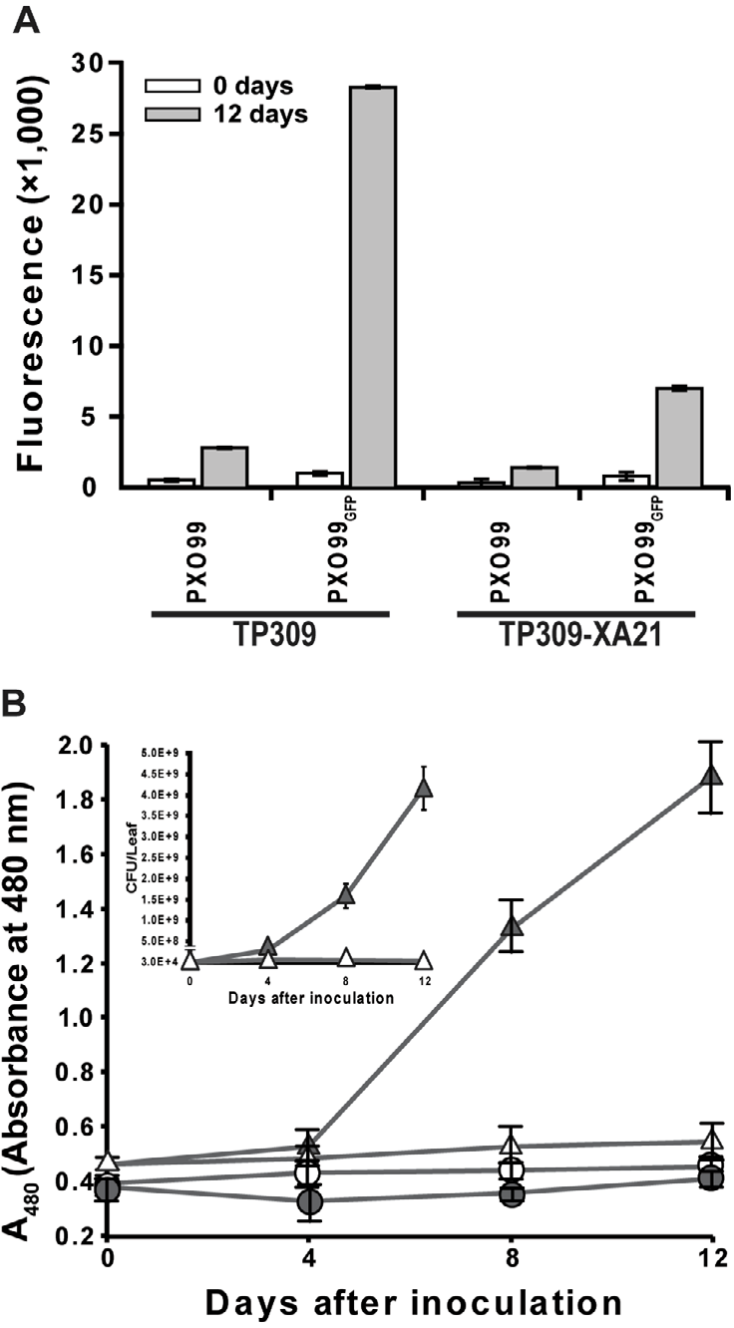
**Quantification of PXO99_GFP _expressing GFP using a fluoremeter or ELISA**. A. Fluorescence of the bacteria recovered from the rice leaves was directly measured with a fluoremeter (excitation, 480 nm; emission, 520 nm). The bars indicate average of the fluorescence of each sample and the error bars are standard deviations from six technical replicates of two biological replicates. B. GFP protein expressed in PXO99_GFP _under host condition were quantified using ELISA, Inoculated bacterial strains, PXO99 (circle) and PXO99_GFP _(triangle), were recovered from the scored rice leaves of TP309 (close) and TP309-XA21 (open) at 0, 4, 8, and 12 DAI. ELISA using anti-GFP polyclonal IgG antibody then carried out. Each value represents averages of the absorbance at 480 nm and standard deviation from three leaves per treatment. Inset is the bacterial growth curve with linear scale. This experiment was repeated three times with more than ten rice leaves.

To examine whether bacterial proliferation can be quantified using fluorescent microscopy, PXO99 wild type and PXO99_GFP _cells were recovered from inoculated rice leaves and measured. Infection of PXO99_GFP _was clearly different between susceptible and resistant rice lines (Fig. 4A). At 12 days after infection, the fluorescence dramatically increased from 983 at 0 days to 28,301 relative fluorescence units (RFU) in the susceptible line, TP309. In the resistant line, TP309-XA21, the fluorescence increased from 780 at day 0 to 7,009 RFU. The non-GFP strain, PXO99, also showed a low level of fluorescence that increased slightly by day 12, which is attributed to (plant) auto-fluorescence and not GFP from a bacterial population. These results suggest that measurements of bacterial multiplication using the PXO99_GFP _strain can replace the conventional colony counting method to screen for susceptible and resistant hosts.

The ELISA (enzyme-linked immunosorbent assay) is widely used to quantify a particular protein. This method has been applied as a diagnostic tool to detect specific pathogen proteins in medicine and plant pathology [29,30]. To quantify the multiplication of the PXO99_GFP _strain in rice, ELISA was carried out with anti-GFP antibody and the results were compared with the populations of the PXO99_GFP _strain at the indicated time points (Fig. 4B). PXO99 and PXO99_GFP _strains were harvested from TP309 and TP309-XA21 at 0, 4, 8, and 12 DAI. While the PXO99 strain showed a background absorbance level at all time points (approximately 0.4 A_480 _(absorbance at 480 nm)), the PXO99_GFP _strain showed a temporal-dependent increase of GFP. The level of GFP expression in the resistant rice line was maintained with a gentle increase until 12 DAI. Interestingly, the GFP in the susceptible rice line, TP309, increased after 4 DAI and reached up to 2.0 A_480 _at 12 DAI. This increase of GFP observed in ELISA correlated well with PXO99_GFP _population levels (inset in Fig. 4B), indicating that the significant increase of GFP in rice is dependent on bacterial multiplication. Because the fluorescent marker approach does not require diluting, incubating, and counting of bacterial cells, the method is 3 days faster than conventional measurement methods using plate assays. Taken together, these results suggest that measurement of the bacterial fluorescence and GFP quantification by ELISA are sensitive and rapid methods to determine growing bacterial population and host resistance compared to conventional methods.

## Conclusion

In this report, we introduce an application of a fluorescent marker system for study of the phytopathogenic bacterium, *X. oryzae *pv. *oryzae*. The fluorescent *X. oryzae *pv. *oryzae *strain, PXO99_GFP_, facilitates detection of the invading pathogen. With this fluorescent marker system, bacterial population can be measured in a day, and resistant/susceptible lines can be screened at 4 DPI. This application, which may be used for other patho-systems, can reduce the time and effort required for bacterial growth determination compared to the conventional method.

## Methods

### Bacteria strains, plants and growth conditions

*Xanthomonas oryzae *pv. *oryzae *Philippine race 6 (PXO99Az kindly provided by Jan Leach and called PXO99 in this study) was used for this study [31]. The bacteria strains and plasmids used in this study are listed in Table 1. Peptone sucrose media [32] containing cephalexin (20 μg/ml) (MP Biomedicals, USA) or cephalexin (20 μg/ml)/kanamycin (50 μg/ml) were used for growing cultures of PXO99 or PXO99_GFP _at 28°C, respectively. *Escherichia coli *strains were cultured in Luria-Bertani (LB) medium at 37°C. For *E. coli*, kanamycin at 50 μg/ml, ampicillin at 100 μg/ml or gentamycin at 25 μg/ml were used for selection of transformants.

**Table 1:** Bacterial strains and plasmids used in this study

Strain or plasmid	Relevant characteristics	Source or reference
*Escherichia coli*		
DH10B	F-*mcrAΔ(mrr-hsdRMS-mcrBC) *φ80/acZΔM15Δ*l*acX74 *deoR recA1 endA1 araΔ*139Δ(*ara*, *leu*)7687*galU galKλrpsL*(Sp^r^)*nupGλtonA*	Gibco BRL

*Xanthomonas oryzae *pv. *oryzae*		
PXO99	Philippine race 6 (PR6) strain, Cp^r^	[31]
PXO99_GFP_	PXO99 with pPROBE-NT*pNm*, Km^r^, Cp^r^	This study

Plasmids		
pPROBE-*gfp*	pBBR1 ori, Km^r^, broad-host range expression vector	[20]
*pPneo-gfp*	*neo *(promoter of neomycin resistant gene) cloned into pPROBE-*gfp*	This study
pML122	OriV, OriT, Gm^r^, pNm (*nptll*), broad-host range expression vector	[21]

Cp^r^: Cephalexin resistance, Sp^r^: Spectinomycin resistance, Km^r^: Kanamycin resistance, Gm^r^: Gentamycin resistance

For inoculation experiments to test for virulence, two different cultivars of the *japonica *rice varieties Taipei 309 (TP309) and Kitaake (Kit) were used. Transgenic mutant lines of the cultivars expressing XA21 protein, TP309-XA21 and Kit-XA21, that are resistant to PXO99 strain expressing AvrXA21 [33] were used for avirulence test. The surface was sterilized in 95% ethanol for 5 min, 40% bleach for 30 min with shaking and then three times rinsed with sterilized water. Rice seeds were germinated for three or four days in water at 37°C in water. The germinated seeds were planted onto soil and grown in greenhouse. Six-week-old rice for TP309 line and five-week-old rice for Kit lines were transferred into growth chamber at least two day prior to inoculation. The growth chamber was set on a 16 h light and 8 h dark photoperiod, a 28/26°C temperature cycle, and 90% humidity.

### Molecular techniques

Standard methods described in Sambrook *et al. *[34] were used for DNA manipulations during plasmid preparations and digestions with restriction enzymes. All enzymes for construction of plasmid were purchased from Invitrogen. DNA and protein concentrations were measured using the Nanodrop^® ^ND-1000 (Bio-Rad, USA). The Cell-Porator™ system (Gibco BRL, USA) was used for *E. coli *(≥ 600 DC) and *X. oryzae *pv. *oryzae *(≥ 700 DC) transformations under the following conditions; booster: 4, capacitance: 330 μF, charge rate: fast, low α.

### Generation of *Xanthomonas oryzae *pv. *oryzae *mutant strain carrying *green fluorescent protein *(*gfp*) gene

Broad-host-range promoterless probe vector, pPROBE-*gfp *[20], was used for generation of *X. oryzae *pv. *oryzae *mutant strain constitutively expressing GFP. Neomycin promoter (approximately 400 nucleotide-lengths) cut out with *Sal *I and *EcoR *I from pML122 vector [21] was inserted into the multi-cloning site of the pPROBE-*gfp *vector linearized with the same enzymes. The construct for constitutively expressing GFP, pP*neo*-*gfp*, was introduced into *E. coli *competent cell, DH10B, and then selected on the LB plates containing 50 μg/ml of kanamycin. The plasmid DNA extracted from the *E. coli *transformants re-introduced into PXO99 competent cells using electroporation method. The PXO99 mutant strain carrying the pP*neo*-*gfp *was (PXO99_GFP_) selected on the PSA plates containing 20 μg/ml of cephalexin and 50 μg/ml of kanamycin. The transformants also confirmed by observation using a Zeiss Axiophot fluorescence microscope (Jena, Germany) equipped with a fluorescein isothiocyanate filter (excitation filter, 450 to 490 nm; emission filter, 520 nm; dichroic mirror, 510 nm).

### Inoculation experiment and bacteria growth *in planta*

*X. oryzae *pv. *oryzae *strains were prepared by culturing on PSA plates containing either cephalexin (for the PXO99) or cephalexin/kanamycin (for the PXO99_GFP_) for three days at 28°C. Rice leaves were inoculated with the scissor clipping method [24], using cells suspended in distilled water at a density approaching 1.0 × 10^8 ^CFU/ml. Lesion lengths were measured 0, 4, 8, and 12 DAI.

*X. oryzae *pv. *oryzae *growth *in planta *was measured using a modified method from a report by Song *et al*. [33]. For establishing growth curves, the inoculated rice leaves were harvested at each time point, immediately sliced into small pieces. Sliced rice leaves were incubated in 1 ml sterile water including 15 μg/ml of cephalexin with shaking for 1 h, and then filtered through two layers of cheesecloth. The filtrates were then plated onto PSA plates with cephalexin for PXO99 strain or cephalexin/kanamycin for PXO99_GFP _strain. Colonies on the plates were counted after three days of incubation at 28°C.

### Microscope

To visualize bacterial infection through leaf veins under microscope, rice leaves were harvested at 12 DAI. To get thin transverse sections (0.1 mm), TP309 and TP309-XA21 leaves inoculated with PXO99_GFP _were cut using a razor blade. The small pieces of leaf sections were placed on a microscope slide, submerged in immersion oil (Cargille lab, USA), covered with a glass slip and sealed with grease. The fluorescence photographs were taken using a Zeiss Axiophot fluorescence microscope (Jena, Germany) fitted with fluorescein isothiocyanate filters (excitation filter, 450 to 490 nm; emission filter, 520 nm; dichroic mirror, 510 nm). The optimal exposure time was 1 sec. Three biological replicates using leaf blades from each of the three different plants were used for all microscopic analyses.

### Fluorimetric measurements of PXO99_GFP _strain

*X. oryzae *pv. *oryzae *strains were inoculated on 6 weeks old TP309 and TP309-XA21 lines, and to quantify GFP using a fluoremeter, *X. oryzae *pv. *oryzae *cells were recovered at 0, and 12 DPI as described in the previous section. The bacteria recovered from the rice leaves were serially diluted until 10^-4 ^with water and 200 μl of cell suspension were transferred in a microtitre plate, MICROTEST™ 96 (Falcon, USA). Fluorescence of the bacteria was directly measured in a fluoremeter, SafireII (Tecan, USA) [35]. GFP fluorescence was measured at a wavelength of 480 nm for excitation and 520 nm for emission, with a bandwidth of 20 nm in both cases.

### Enzyme-Linked ImmunoSorbant Assay

Leaves were collected from TP309 and TP309-XA21 at 0, 4, 8, and 12 DAI by PXO99 or PXO99_GFP _strain. The inoculated rice leaves were harvested at each time point, immediately sliced into small pieces, incubated in 1 ml sterile water including 15 μg/ml of cephalexin with shaking for 1 h, and then filtered through two layers of cheesecloth. The filtrates were precipitated by centrifugation at 15,000 g for 15 min, and the pellets were dissolved in 100 μl of lysis buffer (100 mM Tris-Cl, pH 8.0, 8 M urea, and 100 mM NaH_2_PO_4_). Micro-ELISA plates (96-well plate, Fisher Scientific, UK) were coated with 200 μl of PXO99 or PXO99_GFP _diluted with carbonate-bicarbonate buffer (0.2 M sodium carbonate-bicarbonate, pH9.4). After overnight incubation at 4°C, plates were washed three times with 0.5% Tween-20 in PBS buffer (10 mM phosphate buffer, pH 7.4, 150 mM NaCl, and 0.1% sodium azide; PBST). Plates were treated with 5% skim milk in PBS and washed three times with PBST buffer. Anti-GFP polyclonal IgG antibody (Santa cruz, USA) of 1:500 dilution was added to each well (100 μl/well) and incubated for 1 h at 37°C. After three washings with PBST, a 1: 5,000 diluted horseradish peroxidase linked anti-rabbit IgG antibody (GE healthcare, UK) was added for 1 h at 37°C. The plates were subsequently washed three times with PBST. To detect peroxidase activity, one OPD (ortho-phylenediamine) tablet (Sigma, USA) and one urea hydrogen peroxidase tablet (Sigma, USA) was dissolved in 20 ml of distilled water. 200 μl of substrate was added to each well and incubate the plate in the dark for 20 min at room temperature followed by addition of 50 μl 3 M HCl to stop the reaction. Absorbance of the plate was measured at 480 nm by an ELISA reader, SafireII (Tecan, USA).

### Ethics

Exempt – animal subjects are not used.

## Competing interests

The authors declare that they have no competing interests.

## Authors' contributions

SWH carried out inoculation experiments including lesion measurement, bacterial growth curve with conventional method, and recovery of bacterial cells and proteins for ELISA and fluorescence. CJP carried out microscope observation and ELISA. SWL generate all bacterial strains for this study and measured fluorescence. SWH, CJP, SWL, and PCR participated in its design and coordination and helped to draft the manuscript. All authors read and approved the final manuscript.

## Supplementary Material

Additional file 1 **Comparison of disease progress in TP309 and TP309-XA21 by inoculation of PXO99 and PXO99GFP**. A. Lesion lengths of PXO99 (circle) and PXO99GFP (triangle) inoculated plants, TP309 (closed) and TP309-XA21 (opened). Each data represents the average and standard deviation that were established from more than ten leaves. B. Plots of PXO99 (circle) and PXO99GFP (triangle) populations at 0, 4, 8, and 12 DAI in TP309 (closed) and TP309-XA21 (opened). Cultured PXO99 and PXO99GFP strains were diluted to 1.0 × 10^8 ^CFU/ml and then inoculated to TP309 and TP309-XA21 rice leaves using scissor clipping method respectively. The data in each time point were calculated from three leaves and repetition three times. Click here for file

Additional file 2 **Measurements of bacterial migration and lesion length in TP309 and TP309-XA21 by inoculation of PXO99GFP strain at 4 DAI**. After culture of PXO99GFP strain on PSA plates containing cephalexin/kanamycin, cells were diluted to 1.0 × 10^8 ^CFU/ml, and then inoculated onto rice leaves of TP309 (susceptible) and TP309-XA21 (resistant) lines using the scissor clipping method. Bacterial migration (grey bar) and lesion length (white bar) were measured from inoculation sites at 4 DAI. Fluorescent bacteria in collected samples were observed with microscope equipped with a fluorescein isothiocyanate filter (excitation filter, 450 to 490 nm; emission filter, 520 nm; dichroic mirror, 510 nm). Each bar represents averages ± standard deviation. The experiments were repeated three times with more than ten rice leaves from three individuals each time. Click here for file
