# Supplementary figures and images for: An efficient method for visualization and growth of fluorescent Xanthomonas oryzae pv. oryzae in planta
Source: BMC Microbiol. 2008 Sep 30;8:164. doi: 10.1186/1471-2180-8-164 (PMC2569045; doi:10.1186/1471-2180-8-164)

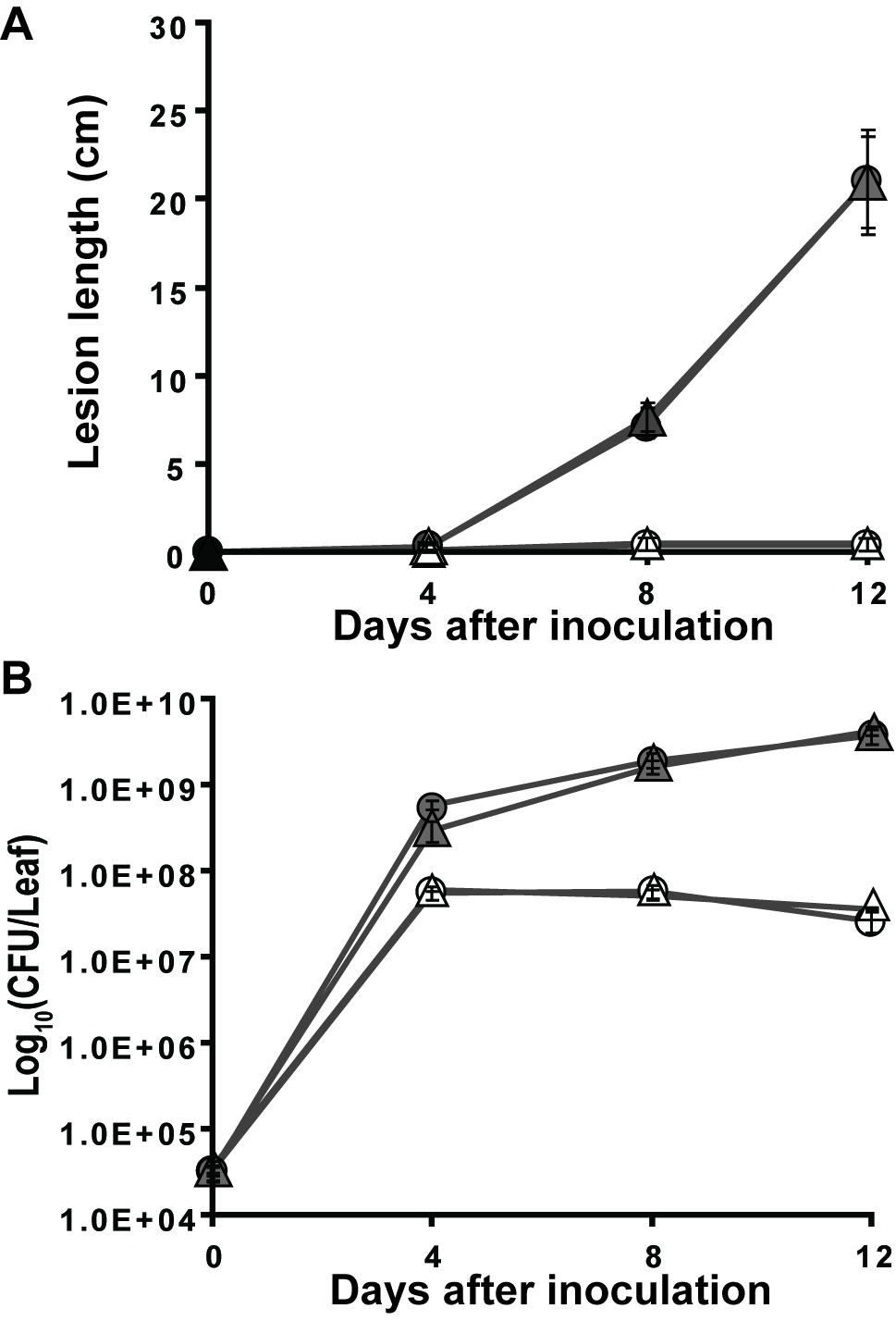

Supplement: Additional file 1 — Comparison of disease progress in TP309 and TP309-XA21 by inoculation of PXO99 and PXO99GFP. A. Lesion lengths of PXO99 (circle) and PXO99GFP (triangle) inoculated plants, TP309 (closed) and TP309-XA21 (opened). Each data represents the average and standard deviation that were established from more than ten leaves. B. Plots of PXO99 (circle) and PXO99GFP (triangle) populations at 0, 4, 8, and 12 DAI in TP309 (closed) and TP309-XA21 (opened). Cultured PXO99 and PXO99GFP strains were diluted to 1.0 × 10^8^ CFU/ml and then inoculated to TP309 and TP309-XA21 rice leaves using scissor clipping method respectively. The data in each time point were calculated from three leaves and repetition three times. [file 1471-2180-8-164-S1.tiff]

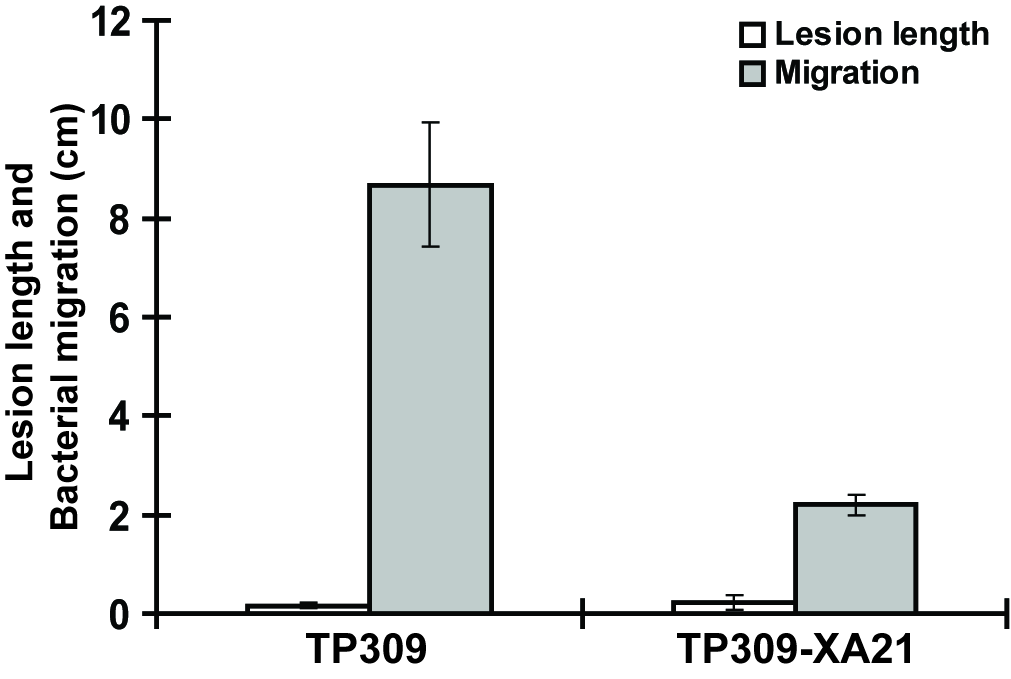

Supplement: Additional file 2 — Measurements of bacterial migration and lesion length in TP309 and TP309-XA21 by inoculation of PXO99GFP strain at 4 DAI. After culture of PXO99GFP strain on PSA plates containing cephalexin/kanamycin, cells were diluted to 1.0 × 10^8^ CFU/ml, and then inoculated onto rice leaves of TP309 (susceptible) and TP309-XA21 (resistant) lines using the scissor clipping method. Bacterial migration (grey bar) and lesion length (white bar) were measured from inoculation sites at 4 DAI. Fluorescent bacteria in collected samples were observed with microscope equipped with a fluorescein isothiocyanate filter (excitation filter, 450 to 490 nm; emission filter, 520 nm; dichroic mirror, 510 nm). Each bar represents averages ± standard deviation. The experiments were repeated three times with more than ten rice leaves from three individuals each time. [file 1471-2180-8-164-S2.tiff]
